# Novel human recombinant antibodies against Mycobacterium tuberculosis antigen 85B

**DOI:** 10.1186/1472-6750-14-68

**Published:** 2014-07-17

**Authors:** Manon Fuchs, Susanne Kämpfer, Saskia Helmsing, Ralf Spallek, Wulf Oehlmann, Wiebke Prilop, Ronald Frank, Stefan Dübel, Mahavir Singh, Michael Hust

**Affiliations:** 1 Technische Universität Braunschweig, Institut für Biochemie und Biotechnologie, Spielmannstr.7, 38106 Braunschweig, Germany; 2 Lionex GmbH, Salzdahlumer Str. 196, 38126 Braunschweig, Germany; 3 Helmholtz Zentrum für Infektionsforschung, Abteilung Chemische Biologie, Inhoffenstr. 7, 38124 Braunschweig, Germany; 4 Leibniz-Institut für Molekulare Pharmakologie, Robert-Roessle-Str. 10, 13125 Berlin, Germany

## Abstract

**Background:**

Tuberculosis is the leading cause of death due to bacterial infections worldwide, mainly caused by *Mycobacterium tuberculosis*. The antigen 85 complex comprises a set of major secreted proteins of *M. tuberculosis*, which are potential biomarkers for diagnostic.

**Results:**

In this work, the first human single chain fragment variable (scFv) antibodies specific for the tuberculosis biomarker 85 B were selected by phage display from naïve antibody gene libraries (HAL7/8). Produced as scFv-Fc in mammalian cells, these antibodies were further characterized and analysed for specificity and applicability in different tuberculosis antigen detection assays. Sandwich detection of recombinant 85 B was successful in enzyme linked immunosorbent assay (ELISA), lateral flow immunoassay and immunoblot. Whereas detection of *M. tuberculosis* cell extracts and culture filtrates was only possible in direct ELISA and immunoblot assays. It was found that the conformation of 85 B, depending on sample treatment, influenced antigen detection.

**Conclusions:**

Recombinant antibodies, selected by phage display, may be applicable for 85 B detection in various assays. These antibodies are candidates for the development of future point of care tuberculosis diagnostic kits. Using 85 B as a biomarker, the antigen conformation influenced by sample treatment is important.

## Background

Approximately 8.7 million people worldwide fell ill with tuberculosis (TB) in 2011 and 1.4 million deaths were reported [1]. Worldwide TB ranks as the second major cause of death from an infectious disease. One third of the world population is estimated to be infected with *M. tuberculosis* (Mtb), yet they remain asymptomatic. This is defined as latent TB infection (LTBI) [2]. Only 66% of the TB-cases worldwide are correctly diagnosed [1]. The gold standard in TB diagnosis remains the preparation of liquid cultures in selective media from sputum or tissue/body fluid specimens [3]. This is followed by further Mtb specific tests or drug susceptibility testing (i.e. nucleic acid amplification tests such as the Gene Xpert MTB/RIF [4]).

Diagnosis of TB in most low- and middle-income countries continues to rely on sputum smear microscopy for acid-fast bacilli (Ziel-Neelsen stain) [1]. This technique detects only 40 – 60% of pulmonary TB cases and is not able to differentiate between Mtb and other ubiquitous mycobacteria [5,6]. This is less sensitive in children, HIV co-infected patients and in patients with extrapulmonary TB [7,8]. The diagnosis of TB in developing countries is limited by equipment and infrastructure [1]. Therefore, in these countries, a simple diagnostic tool without the need for sophisticated instruments is needed. Accuracy, simplicity, affordability and technical robustness are important factors for a point of care (POC) TB-test. The main advantage of this is a rapid diagnosis, which allows initiation of treatment while the patient is still accessible [9]. Several anti-tuberculosis antibody detection systems are available. However the WHO recommended against the use of these assays because of their lack of sensitivity and specificity [10]. Direct detection of Mtb antigens in human specimens would allow specific diagnosis of active TB to be made, independent of the host’s immune response. Furthermore, the use of specific antibodies to Mtb antigens in a lateral flow immuno assay (LFIA) would potentially provide a rapid POC test in a cost effective, easy-to-use format. Potential target antigens for POC TB-detection in human samples should be selected by the following criteria: substantial expression by bacteria *in vivo*, presence in the extracellular environment or on the mycobacterial cell wall, and resistance to degradation by host enzymes [11]. The 85 complex is a major secretion product of Mtb [12,13] which comprises three variant 85 proteins (A, B and C) that are encoded by three different genes (fbpA, fbpB and fbpC2, [14]). Antigens 85 A, 85 B and 85 C possess mycoyltransferase activity and are involved in the formation of trehalose monomycolate (TMM) and trehalose dimycolate (TDM), which are components of the mycobacterial cell wall [15,16]. Besides the 85 complex there is another 85 protein, known as 85 D or FbpC1. 85 D shows structural similarity to the 85 complex proteins, yet does not possess mycoyltransferase activity [15] and its function remains unknown. Disruption of fbpA results in the inability of Mtb to replicate within macrophages, indicating a key role in virulence [17]. Furthermore, it was shown that 85 complex proteins interact with gelatine-binding sites of human fibronectin, enhancing complement-mediated phagocytosis by macrophages [18-20]. Antibodies specific for only one of the 85 proteins would allow for further investigation of their individual roles in the pathogenesis of TB. However only antibodies cross reacting with all other 85 complex proteins have been reported so far [21-23].

The presence of the 85 complex was demonstrated in human serum [24], urine [20], cerebrospinal fluid [25] and sputum [26] of TB infected individuals. This makes it a prominent marker for TB. Although other mycobacteria express similar 85 complex proteins, these antigens could be used as TB markers in combination with other antigens to enhance specificity.

For the future development of diagnostic assays, recombinant antibodies generated by phage display are an alternative to polyclonal and monoclonal antibodies [27-32]. Naïve antibody libraries allow generation of antibodies to epitopes which are not recognized by any immune system. In addition, recombinant human antibodies could be used in therapeutic applications. In this work, single chain Fv (scFv) were isolated from a human naïve antibody gene library using phage display [33] and produced as scFv-Fc (yumab) [34]. These antibodies were further characterized and analysed for their suitability in an antigen detection assay targeting Mtb 85 B.

## Results

### Selection of human antibodies against 85 B

For antibody selection, panning was performed on immobilized 85 B followed by a screening ELISA using soluble scFv (see Additional file 1: Figure S1). Finally, five unique scFv were confirmed by DNA sequencing. The corresponding human germline sequences according to VBASE2 [35] are displayed in Table 1. All α-85 B antibodies contain a variable gene segment of the heavy chain (HV) of subfamily V_H_3. Four binders have a lambda light chain (V_L_λ) and one has a kappa light chain (V_L_κ). All V_L_λ clones have a joining segment of the heavy chain (HJ) of subfamily J4, a joining segment of the light chain (LJ) of subfamily J3, and a variable gene segment of the heavy chain (HV) of subfamily V_H_3 gene 30.

**Table 1:** Comparison of heavy and light chain gene segments of α-85 B antibodies

	**Heavy chain**			**Light chain**	
**Clone**	**HV**	**D**	**HJ**	**LV**	**LJ**
MFU50-A10	IGHV3-30*04	IGHD6-19*01	IGHJ4*02	IGLV8-61*01	IGLJ3*02
MFU50-C10	IGHV3	IGHD2-15*01	IGHJ6*02	IGKV1-12*01	IGKJ2*01
MFU50-D4	IGHV3-30*18	IGHD3-3*02	IGHJ4*02	IGLV2-14*01	IGLJ3*01
MFU50-D7	IGHV3-30*04	IGHD4-17*01	IGHJ4*02	IGLV3-21*02	IGLJ3*02
MFU50-E2	IGHV3-30*03	IGHD6-13*01	IGHJ4*02	IGLV7-43*01	IGLJ3*02

*Abbreviations: HV* variable (V) gene segment of the heavy chain, *D* diversity gene segment, *HJ* joining (J) gene segment of the heavy chain, *LV* variable gene segment of the light chain, *LJ* joining gene segment of the light chain.

### Cloning and production of scFv-Fc (Yumabs)

The scFv were subcloned into pCSE2.5-hIgG1-Fc-XP [34], produced in 50 mL scale and purified from the culture supernatant via Protein A. The purified scFv-Fc (Yumabs) were analysed by SDS-PAGE, silver staining, α-human IgG(Fc) immunoblot and reducing gel analysis via Tape Station. No degradation was detected (data not shown). The purity of the obtained antibody preparations was determined to 93.4 – 96.9%.

### Validation of antigen binding

**Figure 1:**
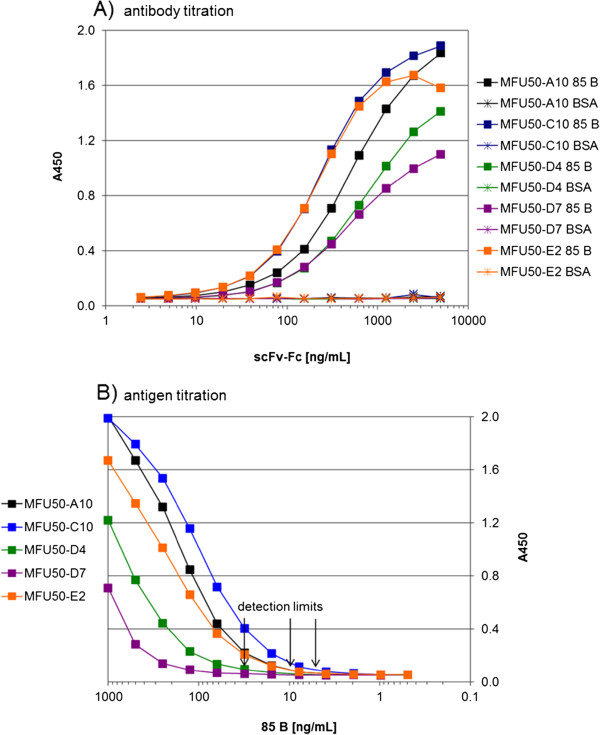
**Analysis of antigen binding. A)** Antibody titration and **B)** antigen titration in ELISA with α-85 B scFv-Fc. A) Dilution series of scFv-Fc were used for detection of directly coated antigen (85 B) or BSA (negative control). Detection of bound scFv-Fc with goat α-human IgG(Fc)-HRP and development with TMB. B) Different dilutions of antigen (85 B) were directly coated to the wells. Antigen detection with a scFv-Fc concentration of half maximal saturation. Detection of bound scFv-Fc with goat α-human IgG(Fc)-HRP, development with TMB. Negative control BSA A450 = 0.05.

The antigen binding of the Yumabs was analysed by titration ELISA (Figure 1A). The antigen binding was confirmed for all Yumabs. Antigen detection limits of the α-85 B scFv-Fc were determined by antigen titration ELISA (Figure 1B). About 5 ng/mL were detected by MFU50-C10, ~ 10 ng/mL by MFU50-A10 and MFU50-E2, and ~ 30 ng/mL by MFU50-D4 and MFU50-D7.

### Epitope mapping

**Figure 2:**
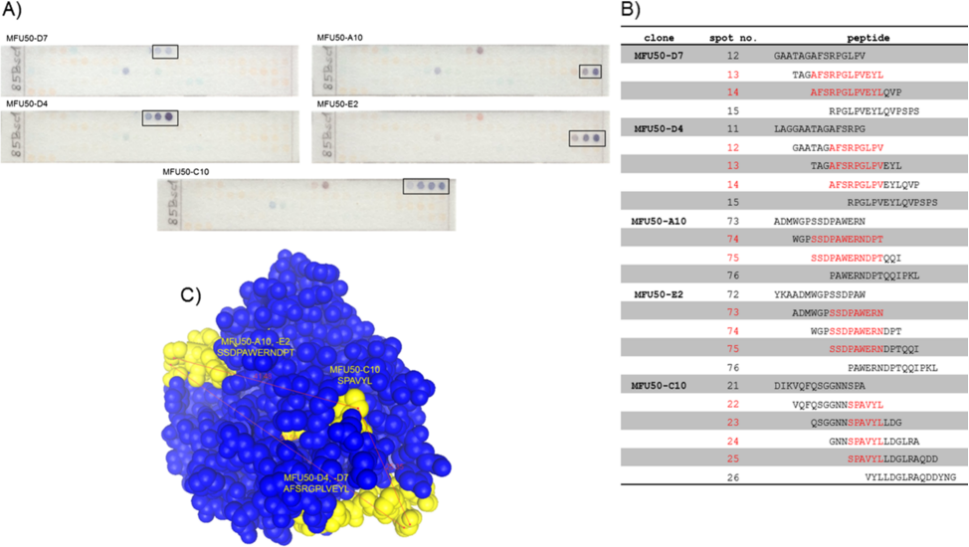
**Analysis of the epitope. A)** Epitope mapping of α-85 B scFv-Fc with PepSpot membrane, **B)** Amino acid sequences of PepSpots and **C)** 3 D structure of 85 B and α-85 B antibody epitopes. A) Membrane was incubated with respectively 5 μg scFv-Fc, detection of bound scFv-Fc with goat α-human IgG(Fc)-HRP, development with TMB. Immunostained spots are circled. No cross reaction of the secondary antibody with the PepSpots was determined (data not shown). B) Amino acid sequences of peptides on 85 B PepSpot membrane. The immunostained spots and identified epitopes are highlighted. C) 3D structure of antigen 85 B, epitopes of α-85 B antibodies. Pdb1f0n (Resolution 1.9 Å, [36]) was modified with 3D molecule viewer (Invitrogen). Only protein chain A is shown, atoms are displayed as space filling balls, epitopes are marked in yellow, distance measurement in red.

To determine whether the antibodies bind to linear or conformational epitopes, the α-85 B scFv-Fc were analysed by immunoblot (data not shown). All antibodies recognized linear epitopes (data not shown). Epitope mapping of the α-85 B scFv-Fc was carried out on PepSpot membranes (Figure 2A). Through the overlap of the peptide sequences the epitopes were determined (Figure 2B). The crystal structure of antigen 85 B was determined by Anderson et al. [36] and the epitopes were visualized on the 3D structure of the antigen (Figure 2C). The distance between epitope regions “AFSRPGPLV(EYL)” and “SPAVYL” was computed to ~ 2.5 nm, between “SPAVYL” and “SSDPAWERN(DPT)” to ~ 4 nm and between “SSDPAWERN(DPT)” and “AFSRPGPLV(EYL)” to ~ 5 nm. Considering the width of an antibody (~4 nm, [37]), sandwich detection was likely targeting epitopes on different sites of the antigen.

### Cross reactions

**Figure 3:**
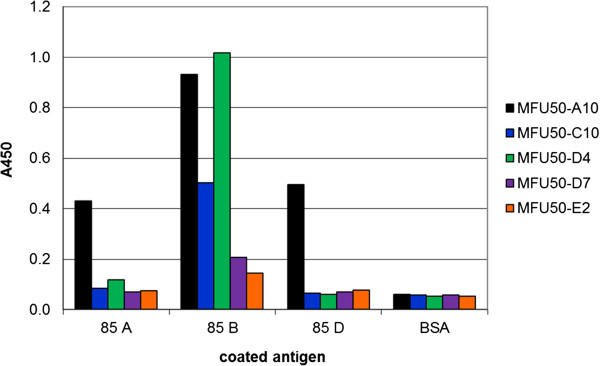
**Cross reactions of α-85 B scFv-Fc with other 85 complex antigens in ELISA.** 100 ng of the antigens were directly coated to the wells. 85 B detection with α-85 B scFv-Fc (using concentrations at half maximal saturation), detection of bound scFv-Fc with goat α-human IgG (Fc)-HRP, development with TMB.

Sequence comparison of the epitopes of the α-85 B antibodies with corresponding sequences of the other 85 complex antigens provided information about possible cross reactions (Table 2). The complete epitope “AFSRPGPLV(EYL)” is present in antigen 85 A and 85 C, but not in 85 D. Homologous regions of the epitopes “SPAVYL” and “SSDPAWERN(DPT)” can be found in 85 A, C, and D. Experimental study of 85 complex cross reactivity was carried out by indirect ELISA (Figure 3). MFU50-A10 reacted with 85 A and 85 D. MFU50-D4 displayed a weak cross reactivity with 85 A as predicted. MFU50-E2 bound antigen 85 B weakly and showed no cross reactions. MFU50-D7 reacted weakly with 85 B. No reaction with the other 85 complex antigens was detected. MFU50-C10 was also not cross reactive. A summary of the expected cross reactions in comparison to determined cross reactions is outlined in Table 2.

**Table 2:** Cross reactions of α-85 B antibodies with other 85 complex antigens

**sequence comparison (corresponding sequences to α-85 B epitopes)**					
Clone	85 A	85 B	85 C*	85 D	Expected cross reaction
MFU50-A10	KEDPAWQRNDPL	SSDPAWERNDPT	SSDPAWKRNDPM	SDPA	85 A, 85 C, 85 D
MFU50-C10	SPALYL	SPAVYL	PHAVYL	PHAVYL	85 A, 85 C, 85 D
MFU50-D4	AFSRPGLPV	AFSRPGLPV	AFSRPGLPV	-	85 A, 85 C
MFU50-D7	AFSRPGLPVEYL	AFSRPGLPVEYL	AFSRPGLPVEYL	-	85 A, 85 C
MFU50-E2	KEDPAWQRN	SSDPAWERN	SSDPAWKRN	SDPA	85 A, 85 C, 85 D
**Reaction with 85 complex antigens in ELISA**					
Clone	85 A	85 B	85 C*	85 D	Determined cross reaction
MFU50-A10	Strong	Strong	-	Strong	85 A, 85 D
MFU50-C10	None	Strong	-	None	None
MFU50-D4	Weak	Strong	-	None	Weak 85 A
MFU50-D7	None	Weak	-	None	None
MFU50-E2	None	Weak	-	None	None

*Purified antigen 85 C was not available for immunological assays, nevertheless it is listed for completeness of the illustration.

### Detection of 85 B in Mtb cell extracts and culture filtrates

**Figure 4:**
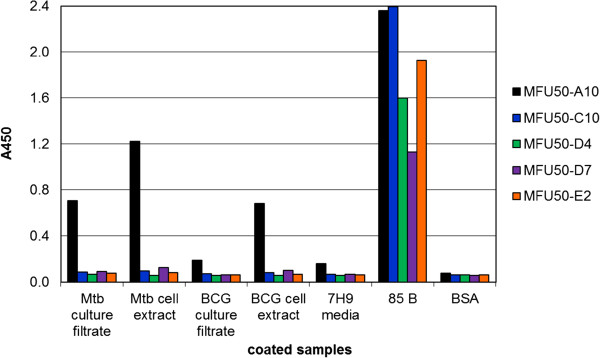
**Reaction of α-85 B scFv-Fc with Mtb/BCG cell extracts and culture filtrates determined by indirect ELISA.** 100 ng/100 μL of the different samples were directly coated to the well, reaction was determined with a scFv-Fc concentration of 2.5 μg/mL. Detection of bound scFv-Fc with goat α-human IgG(Fc)-HRP. Development with TMB.

The antibody binding to Mtb/BCG cell extracts and culture filtrates was analysed by indirect ELISA (Figure 4). In this assay only MFU50-A10 reacted with Mtb culture filtrate and Mtb/BCG cell extracts. The other antibodies bound only to recombinant 85 B. A weak cross reaction of MFU50-A10 with 7H9 medium was detected.

### Development of a sandwich ELISA

**Figure 5:**
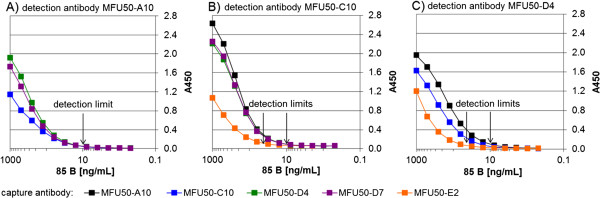
**A-85 B sandwich antigen titration ELISA.** 100 ng of capture scFv-Fc were directly coated to the wells. After blocking different dilutions of antigen were applied and incubated. Detection of bound antigen with: **A)** MFU50-A10-scFv-Fc-HRP at ~0.05 μg/mL and development with TMB. Negative control (BSA) A_450_ = 0.02, **B)** MFU50-C10-scFv-Fc-HRP at ~0.05 μg/mL and development with TMB. Negative control (BSA) A_450_ = 0.05, **C)** MFU50-D4-scFv-Fc-HRP at ~0.05 μg/mL and development with TMB. Negative control (BSA) A_450_ = 0.02.

**Figure 6:**
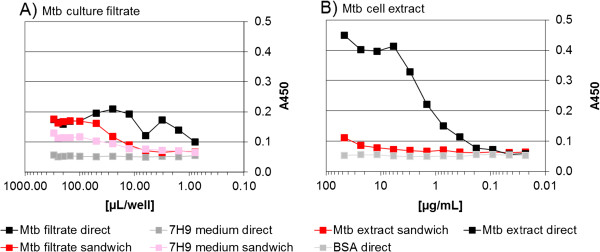
**Mtb culture filtrate and Mtb cell extract titration in α-85 B direct and sandwich ELISA. A)** various volumes of Mtb culture filtrate or **B)** different dilutions of Mtb cell extract were directly coated to the wells. After blocking antigen was detected using MFU50-C10-scFv-Fc-HRP at ~ 0.05 μg/mL and development with TMB. Sandwich ELISA: 100 ng of capture scFv-Fc MFU50-A10 were directly coated to the wells, after blocking A) various volumes of Mtb culture filtrate or B) different dilutions of Mtb cell extract were applied and incubated. Detection of bound antigen with MFU50-C10-scFv-Fc-HRP at ~0.05 μg/mL and development with TMB. Negative controls: BSA, M. smegmatis cell extract, M. vaccae cell extract all A450 = 0.05.

Due to the availability of five different antibodies with three different epitopes on the target antigen a sandwich α-85 B assay was performed. Therefore MFU50-A10 (epitope “SSDPAWERNDPT”), MFU50-C10 (epitope “SPAVYL”) and MFU50-D4 (epitope “AFSRPGLPV”) scFv-Fc were conjugated to HRP. Sandwich ELISA detection of the antigen was carried out with MFU50-A10-HRP, MFU50-C10-HRP or MFU50-D4-HRP as detection antibodies. Capturing was conducted with corresponding antibodies recognizing different epitopes (than the detection antibody) on the target (Figure 5A, B, C). Blocking reagent only was used as a negative control (A_450_ = 0.02 – 0.05). Sandwich detection of the recombinant antigen was successful with all combinations. The antigen detection limit for capturing with MFU50-A10, MFU50-C10, MFU50-D4 or MFU50-D7 was determined to ~ 10 ng/mL, independent from the detection antibody. Capturing with MFU50-E2 resulted in a detection limit of ~ 25 ng/mL. The most suitable combination was obtained with MFU50-A10 as the capture antibody and MFU50-C10-HRP as detection antibody-conjugate. Mtb culture filtrate (Mtb cultivated in 7H9 + ADC + Tween for 3 months at 37°C) was analysed by α-85 B sandwich titration ELISA (Figure 6A, red curve). This resulted in weak binding barely distinguishable from the medium control (Figure 6A, pink curve). Mtb culture filtrate was further analysed by direct α-85 B ELISA (Figure 6A, black curve). In this assay there was no increase of the signal by increasing of sample volume. Additionally, Mtb cell extract was analysed by α-85 B sandwich titration ELISA (Figure 6B, red curve). This showed no binding distinguishable from the negative control except for the highest concentration (Figure 6B, grey curve). However, in a direct α-85 B ELISA specific binding to Mtb cell extract was observed (Figure 6B, black curve).

### Effect of sample treatment on 85 B detection

**Figure 7:**
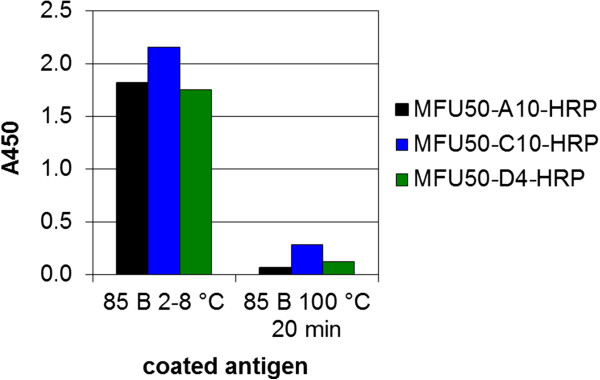
**Detection of boiled 85 B by α-85 B direct ELISA.** 100 ng of antigens in PBS (boiled for 20 min at 100°C or directly taken from storage at 2 – 8°C) were coated to the wells. After blocking bound antigen was detected using MFU50-A10/-C10/-D4-scFv-Fc-HRP at ~0.05 μg/mL and development with TMB. Negative control BSA A450 = 0.02.

**Figure 8:**
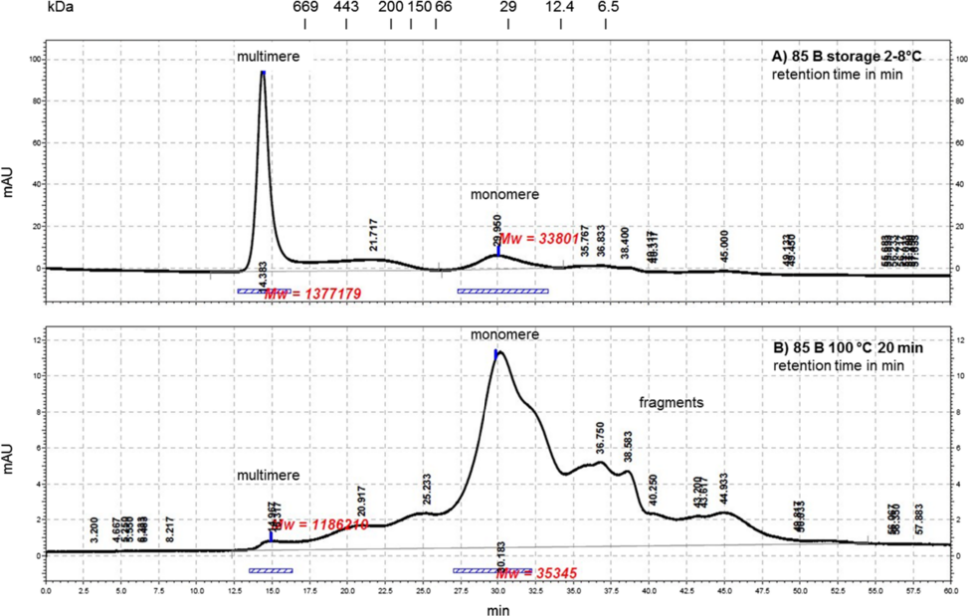
**Analytical SEC of antigen 85 B (batch 09-2/1). A)** Sample stored at 2 – 8°C, **B)** sample boiled for 20 min at 100°C. 500 μL of sample were applied to Superdex 200 10/300 GL column. Running buffer was PBS pH 7.4. Standards: 1. 669 kDa 17 min, 2. 443 kDa 20 min, 3. 200 kDa 23 min, 4. 150 kDa 24 min, 5. 66 kDa 26 min, 6. 29 kDa 31 min, 7. 12.4 kDa 34 min, 8. 6.5 kDa 37 min.

**Figure 9:**
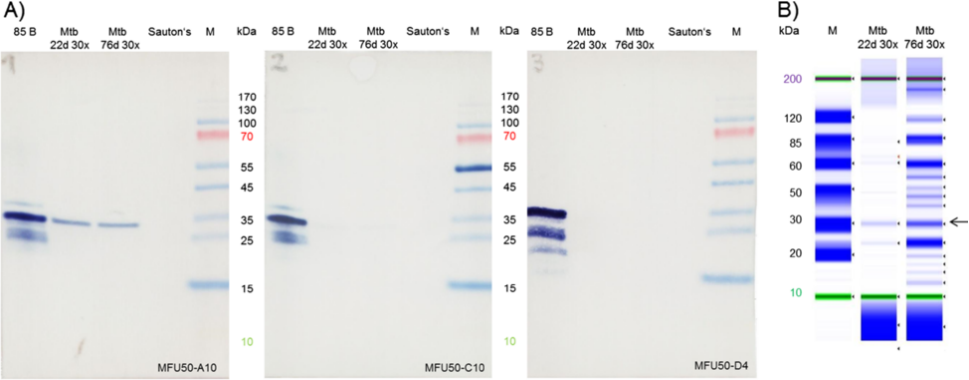
**Detection of 85 B multimers in α-85 B sandwich ELISA. 100 ng of capture scFv-Fc were coated to the wells.** After blocking 100 ng of 85 B in PBS (untreated or boiled for 20 min at 100°C) were applied to the wells and incubated. Bound antigen was detected using **A)** MFU50-A10 **B)** MFU50-C10 and **C)** MFU50-D4-scFv-Fc-HRP at ~0.05 μg/mL and development with TMB. Negative control BSA A_450_ = 0.05. Multimer-detection is indicated by arrows.

The failed detection of 85 B in Mtb cell extract in sandwich ELISA compared to the successful detection in direct ELISA was further analysed. It was hypothesized that the sample treatment, especially autoclaving, was the cause for differing antigen detection. Cell extracts were prepared by autoclaving at 121°C for 20 min. The influence of sample pretreatment by heat on 85 B detection was investigated by direct ELISA (Figure 7). When 85 B was boiled, antigen binding was reduced. Further examination of the susceptibility of 85 B to heat was carried out by analytical SEC. The theoretical molecular mass of an 85 B monomer was computed with the ExPASy prot param tool [38] to 34.6 kDa. The untreated sample of 85 B displayed a small monomer peak at 33.8 kDa and a dominant multimer peak with a molecular mass out of the measurement range but definitely greater than 669 kDa (Figure 8A). In comparison the boiled 85 B sample offered a dominant peak at the size of a monomer (34.6 kDa), multiple peaks with lower molecular mass and a few non-dominant multimer peaks (Figure 8B). Interestingly, aggregation was greater in the untreated sample than in the boiled sample. It was concluded that the aggregation improved sandwich detection, and if multimers were detected by sandwich ELISA, the use of the same antibody for capturing and detection should be possible. Experimental study by sandwich ELISA (Figure 9) verified this assumption. In the untreated as well as in the boiled sample, sandwich detection with only one antibody was possible (Figure 9, arrows).

### Development of a lateral flow immuno assay

**Figure 10:**
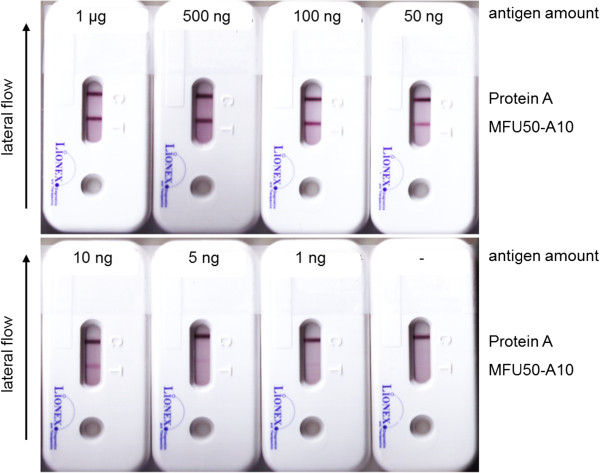
**A-85 B sandwich LFIA, determination of detection limit.** 100 μL sample (7H9 medium spiked with 85 B) + 50 μL 2.2 mM borax pH 8.8 containing 8% BSA were applied on sample pad, read out after 20 min.

To develop a Lateral Flow Immuno Assay (LFIA) all α-85 B scFv-Fc were conjugated to 40 nm colloidal gold. Sandwich 85 B detection was performed with all available antibodies for capturing and all antibody-gold conjugates for detection. The most suitable combination, MFU50-A10 as capture antibody and MFU50-D4-gold as detection antibody, was further analysed. A procedure that allowed sensitive antigen detection and low background combined with good feasibility was developed (data not shown). The 85 B detection limit was determined by sandwich LFIA to ≤ 5 ng/mL (Figure 10). Mtb cell extract or culture filtrate (concentrated and unconcentrated) were analysed by α-85 B sandwich LFIA. Unfortunately, 85 B was not detectable in any sample in this assay.

### Development of an immunoblot assay for the detection of 85B

**Figure 11:**
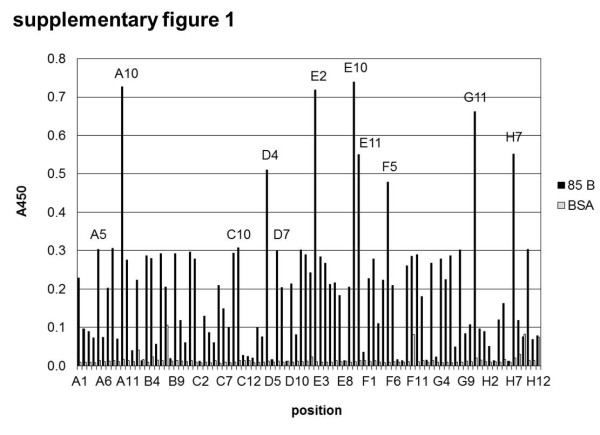
**Detection of 85B by immunoblot. A)** A-85 B immunoblot and **B)** Tape Station analysis of concentrated Mtb culture filtrates (22 d and 76 d at 37°C). A) 100 ng of recombinant 85 B, 25 μL of concentrated Mtb culture filtrates (after 22 or 76 days at 37°C in Sauton’s medium) and 8 μL of marker (M) were separated on reducing 12% SDS-PAGE and electro blotted to a PVDF membrane. After blocking, blots were incubated with scFv-Fc-HRP, development with TMB. B) Samples were directly analysed by reducing gel analysis via Tape Station.

Sandwich detection of antigen 85 B in Mtb culture filtrates and cell extracts was complex in previously described sandwich ELISA and LFIA. As an alternative a direct α-85 B immunoblot assay was developed to avoid the challenge of sample pretreatment in sandwich detection. Furthermore, Mtb was cultured in Sauton’s minimal medium and culture filtrates were concentrated to increase the antigen quantity. Weekly samples were taken, concentrated 20 to 30 fold and analysed by reducing gel analysis via Tape Station and α-85 B immunoblot. No 85 B was detected in 7 and 16 days old cultures by either means (data not shown). After 22 days a protein band at ~30 kDa (according to Tape Station analysis, Figure 11B) was recognized by MFU50-A10 in immunoblot (Figure 11A). Additionally, 85 B expression was found in 61 and 70 days old cultures (data not shown). The Tape Station and α-85 B immunoblot results for 22 and 76 days old cultures are given in Figure 11.

## Discussion

Tuberculosis is the leading cause of death due to bacterial infections worldwide [1]. State of the art TB detection is mostly time consuming or needs expensive infrastructure [39]. In developing countries, a rapid, cheap and easy to use POC TB test is needed [9]. The WHO does not recommend the detection of antibodies against Mtb [10]. The only available assay for direct Mtb antigen detection is that against mycobacterial Lipoarabinomannan (LAM). However this test displays poor sensitivity apart from in patients with advanced HIV/AIDS [40].

In this work, we isolated and characterized specific antibodies against Mtb antigen 85 B. These antibodies were proved suitable for 85 B sandwich detection in ELISA and LFIA. In addition, an Mtb immunoblot assay was developed.

Five unique antibodies were selected from the human naïve libraries HAL7/8 [33]. The examined antibodies displayed homogeneity in the subfamily of the variable gene segment of the heavy chain (all V_H_3). This correlates with the overrepresentation of V_H_1 and V_H_3 in the HAL7/8 libraries [41] and *in vivo*[42]. Four antibodies have a lambda light chain, one a kappa light chain. According to Løset et al. [43] lambda scFv are expressed in higher yields in *E. coli* compared to kappa scFv, which may lead to an advantage in phage display [33,41]. The antibody MFU50-C10 has a germline combination of V_H_3 and V_κ_1, which is common *in vivo* and in naïve libraries [33,42,44]. The lambda germline sequences present in the other antibodies are from the subfamilies 2, 3, 7 and 8. Subfamilies Vλ1,2 and 3 are dominant *in vivo*, whereupon Vλ7 and 8 are rare *in vivo*[42,44]. In the HAL7 library Vλ7 and 8 are more frequent than *in vivo*[33].

Ferrara et al. [22] selected 48 antibodies directed against the Mtb 85 complex in a combination of yeast and phage display. None of these antibodies were specific for an individual 85 antigen but, cross reacted with all other 85 complex proteins. Landowski et al. [45] isolated an α-85 B chicken IgY which cross reacted with 85 complex proteins. Drowart et al. [21] generated seven monoclonal α-85 complex antibodies, which all cross reacted with other mycobacterial species however none were specific for antigen 85 B. This study shows the isolation of the first human recombinant antibodies (MFU50-C10, MFU50-D7 and MFU50-E2) specific for antigen 85 B. Furthermore four α-85 A antibodies (MFU12-D8, MFU53-A3, MFU53-F3 and MFU53-G2) and three α-85 D antibodies (MFU51-A6, MFU51-B10 and MFU53-C2) were generated (unpublished data). MFU12 -D8 was found to be specific for antigen 85 A and MFU51-B10 was specific for 85 D. These antibodies allow for the discrimination between individual components of the 85 complex. Antibody phage display using human naive antibody gene libraries allows for the selection against non-immunogenic proteins and epitopes. We propose these epitopes, which would allow discrimination between the different 85 proteins, are not immunogenic. We propose this because of the fact that no antibodies, specific for one component of the 85 complex, were isolated by hybridoma technology [21,23].

Easy applicability to *in vitro* assays was showed in this study by conjugation to colloidal gold or HRP. Due to the recombinant nature of the explored antibodies, they can easily be altered to different formats, fused to different Fc-parts [34] or fused to markers such as green fluorescent protein (GFP, [46]) for *in vivo* experiments. Thereby the role of particular 85 proteins in Mtb cell wall biosynthesis and evasion of the host’s immune response may be investigated.

All antibodies generated in this study recognized continuous sequences of the antigen, and the corresponding epitopes were determined by screening overlapping peptides immobilized on a cellulose membrane. MFU50-D4 recognized the epitope “AFSRPGLPVEYL” and MFU50-D7 recognized the epitope “AFSRPGLPV”. This epitope region seems to be a potent T cell antigen since synthetic peptides including this sequence were found to induce cytokine release or immune cell proliferation in peripheral blood of individuals with varying TB status [47-50]. However only weak human B cell responses against these peptides were reported [49,51]. Interestingly, no antibodies against this epitope were generated by immunization. In this work, human antibodies were successfully generated by screening phage display libraries, fortifying the advantage of phage display technology over conventional immunization methods. Protein sequence comparison of the 85 complex proteins revealed the presence of the epitope in antigen 85 A and 85 C, suggesting cross reactivity. In ELISA and immunoblot analysis MFU50-D7 showed no 85 A binding, however MFU50-D4 showed slight cross reactivity with 85 A. During this study 85 C was not available for examination. Furthermore, a protein blast search (BLASTP, [52]) disclosed the existence of the entire epitope in several different mycobacterial strains (i.e. *M. bovis, M. ulcerans, M. marinum, M. smegmatis, M. vaccae, etc.*), suggesting cross reactivity.

**Figure 12:**
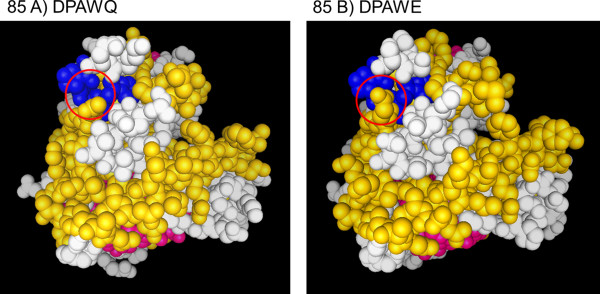
**3 D structures of antigens A) 85 A and B) 85 B around the amino acid sequence “DPAW”.** A) Pdb1sfr (Resolution 2.7 Å, [75]) and B) Pdb1f0n (Resolution 1.9 Å, [36]) were modified with 3D molecule viewer (Invitrogen). Respectively only protein chain A is shown, atoms are displayed as space filling balls, “DPAW” is marked in blue, glutamic acid in 85 B is encircled in comparison to glutamine in 85 A.

The α-85 B antibodies MFU50-A10 and MFU50-E2 recognize the epitopes “SSDPAWERNDPT” and “SSDPAWERN” respectively. Landowski et al. [45] generated an oligoclonal chicken IgY antibody against the peptide “SSDPAWERNDPT” (epitope ID 60953, IEDB) and demonstrated 55% sensitivity and 85% specificity for detection of circulating 85 B in human blood by an immunoblot approach. Shen et al. [51] reported the synthetic peptide “GPSSDPAWERNDPTQ QIPKL” (epitope ID 21797, IEDB) was recognized by sera (IgG) of TB + individuals. Similar peptides containing the epitope “SSDPAWERNDPT” were found to induce cytokine release or T cell proliferation in various assays with samples of TB + individuals [49,50,53-55]. The entire amino acid sequence “SSDPAWERNDPT” is present only in 85 B of *M. tuberculosis* and *M. bovis* BCG. Homologous sequences in Mtb 85 A or 85 C and in 85 complex proteins from other mycobacterial species (identified by BLASTP) differed primarily by substitution of one amino acid. Cross reactivity of MFU50-A10 with Mtb 85 A and *M. bovis* BCG cell extract and culture filtrate was detected by indirect ELISA and immunoblot. Landowski et al. [45] found a “SSDPAWERNDPT” specific chicken IgY antibody to be cross reactive with Mtb 85 A and 85 C as well as 85 complex proteins of *M. avium*. The reaction of MFU50-A10 with 85 A, B and D indicates a possible smaller epitope than determined by epitope mapping. The amino acid sequence “DPA” is present in antigen 85 A, B and C, but the adjacent amino acids differ. In 85 B the “DPA” is flanked by serine on the N-terminal site and tryptophan on the C-terminal site (“SDPAWE”). In 85 A glutamic acid is N-terminal and tryptophan C-terminal (“EDPAWQ”). In 85 D serine is N-terminal and alanine C-terminal (“SDPAAM”). It seems possible that the epitope of MFU50-A10 is “SDPAW” or “DPAWE”, which would mean that two amino acids are different in 85 A or 85 D respectively. This would explain the weaker recognition of 85 A and 85 D. Furthermore, the comparison of the 3D structures of antigen 85 A and 85 B (Figure 12) revealed that the glutamic acid in 85 B forms a pin structure in front of the “DPAW” region. This formation is missing in 85 A, instead of glutamic acid there is glutamine. Glutamic acid can mediate strong electrostatic attractions and hydrogen bridges through the loaded carboxylgroup, whereupon glutamine is uncharged and can only mediate hydrogen bridges through the amino- and the ketogroup [56]. Thus, it is reasoned that the glutamic acid pin structure is needed structurally for correct docking, and physico-chemically for full strength binding to the antigen.

MFU50-C10 recognizes the epitope “SPAVYL”, which is close to the suggested active site of antigen 85 B [36], offering a possible inhibitory effect by steric hindrance [57]. Synthetic peptides including this epitope were found to induce cytokine release and T cell proliferation in peripheral blood mononuclear cells of TB + humans [49,50,54,58] and antibodies against these peptides were detected in sera of TB + individuals [51,59]. Sequence comparison of Mtb 85 complex proteins revealed no homology in this area. According to BLASTP equivalent sequences are present in several other mycobacterial species (i.e. *M. bovis*, *M. marinum*, *M. leprae*, *M. vaccae*, *M. ulcerans*, *M. avium*, etc.). No cross reactivity with 85 A and 85 D was detected by indirect ELISA and immunoblot, suggesting specificity for antigen 85 B. Surprisingly, no cross reactions with *M. bovis* BCG cell extract and culture filtrate were detected in an indirect ELISA.

Sandwich ELISA detection of recombinant 85 B with the α-85 B Yumabs was successful in all performed assays. The most suitable combination was capturing with MFU50-A10 and detection with MFU50-C10-HRP, reaching a detection limit of ~10 ng/mL. Sandwich LFIA detection of recombinant 85 B was successful capturing with MFU50-A10 and detecting with MFU50-D4-gold. A detection limit of 5 ng/strip (0.03 ng/mL) was evaluated. There are no published reports on single antigen 85 B sandwich detection, only whole 85 complex sandwich assays [20,22-24,39,45,60].

Sandwich detection of recombinant 85 B was enhanced by multimeric antigen conformation, and reduced by monomeric antigen conformation. The small size of the antigen (~5 nm diameter, [36]) may be responsible for this finding. Considering the length of a human scFv of ~ 4.5 nm [37] and the Fc-mediated homodimerisation of scFv-Fc fusions [34,61], sterical inhibition by the capture antibody is possible. The commercially available immunochromatographic assays for MPT64 antigen detection in Mtb cultures are sandwich assays with monoclonal antibodies [62]. The Capilia TB Test Kit [63] uses only one monoclonal antibody for a sandwich assay, indicating multimer detection. Recombinant antigen MPT64 showed multimerization by disulphide bonds in two unrelated studies [64,65] and no difference in immunogenicity compared to native antigen [64,66]. These findings imply multimerization of native MPT64 and that these aggregates are detected in sandwich assays. Detection of 85 B in culture filtrates was only possible with MFU50-A10 in direct ELISA and immunoblot. MFU50-A10 was cross reactive with 85 A and 85 D in ELISA and immunoblotting. It has been reported to cross react with 85 C by Landowski et al. [45].

## Conclusions

This work identifies the first recombinant human scFv antibodies specific for antigen 85 B selected by phage display from naïve antibody gene libraries (HAL7/8). These antibodies allow the discrimination between 85 complex proteins, and showed suitability for the establishment of different assays for Mtb 85 B detection. Antigen conformation influenced by sample treatment was shown to be important. The presented antibodies are candidates for the future development of a POC TB diagnostic kit. However, for this purpose an affinity maturation of the antibodies would be necessary to improve the sensitivity.

## Material and methods

All chemicals used were p.a. purity grade and purchased from Sigma Aldrich, Merck or Carl Roth (all located in Germany) or as indicated otherwise.

### Antigen purification

Genes fbpA, B, D coding for proteins of antigen 85 complex of *M. tuberculosis* were amplified by PCR and inserted into pET expression vectors (Novagen, Germany) by site directed cloning (Table 3).

**Table 3:** Cloned genes of M. tuberculosis antigen 85 complex and properties of the recombinant gene products

**Gene**	**Vector**	**Gene product**		
		Antigen	Signal sequence	His-tag
*fbpA* (Rv3804c)	pET26b	85 A	No	N-terminal
*fbpB* (Rv1886c)	pET22b	85 B	Yes	no
*fbpD* (Rv3803c)	pET28c	85 D	Yes	N-terminal

Exchanges L78Q and S196T within the derived amino acid sequence of 85B, and exchange F54L within 85C were detected by DNA sequencing.

Recombinant 85A was produced in *E. coli* strain BL21(DE3) [pLysS] (Novagen, Germany) by growth in LB [67] containing 400 μg/mL ampicillin and 34 μg/mL chloramphenicol. Expression was induced by addition of 1 mM IPTG at mid-log-phase. Cells were harvested 4 hours after induction. 85 B and 85 C were produced using BL21(DE3) (Novagen, Germany) as host strains grown in LB containing 50 μg/mL kanamycin. After induction with 1 mM IPTG at mid-log-phase, cells were incubated overnight and then harvested.

### 85 B purification

Cells were suspended in 10 mM tris/HCl, pH 8.0 containing 1% Triton X-100 and 10 mM EDTA (10 mL per g wet weight). After cell disruption by sonification and centrifugation the pellet was washed once using the same buffer. The pellet was denatured in 20 mM tris/HCl, pH 8.0, 1% Triton X-100 buffer containing 8 M urea and 2 mM DTT.

After centrifugation the supernatant containing the denatured 85 B antigen was bound on a Q-Sepharose High Performance column (GE Healthcare) and eluted by a linear gradient from 0 to 500 mM NaCl. Fractions containing 85 B antigen were pooled and underwent a buffer exchange step on a Sephadex G25 fine column (GE Healthcare) into the buffer described above without Triton X-100. The protein containing pool was subjected to another chromatography step on a Q-Sepharose High Performance column (GE Healthcare). Again protein elution was performed by a linear gradient from 0 – 500 mM NaCl. Pure antigen 85 B containing fractions were pooled and underwent a final refolding and buffer exchange step on a Sephadex G25 fine column (GE Healthcare) into 10 mM NH_4_HCO_3_, 100 μM DTT, pH 7,9.

The final protein preparation is a mixture of protein containing the signal sequence and lacking the signal sequence. Approximately 90% of the protein shows the signal sequence and approximately 10% lacks the signal sequence.

### 85 A purification

Cells were suspended in 20 mM tris/HCl, 100 mM NaCl, pH 8.0 (10 mL per gram wet weight). After cell disruption by sonification and centrifugation, imidazole was added to the supernatant to a final concentration of 5 mM. This solution was applied on a Ni-NTA Superflow column (GE Healthcare) and eluted by a linear gradient from 0 to 500 mM imidazole. Fractions containing 85 A antigen were pooled and underwent a buffer exchange step on a Sephadex G25 fine column (GE Healthcare) into 20 mM tris/HCl, pH 8.0. Chromatography using a Q-Sepharose High Performance column (GE Healthcare) was performed on the pooled protein sample. Protein elution was performed using a linear gradient from 0 – 500 mM NaCl. Pure antigen 85 A containing fractions were pooled and underwent a final buffer exchange step on a Sephadex G25 fine column (GE Healthcare) into 10 mM NH_4_HCO_3_, pH 7.9.

### 85 D purification

Cells were suspended in 20 mM tris/HCl, 100 mM NaCl, pH 8.0 containing 1% Triton X-100 (10 mL per gram wet weight). After cell disruption by sonification and centrifugation the pellet was washed once using 20 mM tris/HCl, 100 mM NaCl, pH 8.0 containing 1% Triton X-100. Afterwards the pellet was denatured in 20 mM tris/HCl, 100 mM NaCl, pH 8.0 containing 8 M urea. After centrifugation the supernatant containing the denatured 85 D antigen underwent a refolding step on a Sephadex G25 fine column (GE Healthcare) into 20 mM tris/HCl, 100 mM NaCl, pH 8.0. The protein peak of this refolding step was collected and then bound on a Q-Sepharose High Performance column (GE Healthcare). Protein elution was performed by a linear gradient from 0 to 300 mM NaCl. Fractions containing pure 85 D antigen were pooled and underwent a buffer exchange step on a Sephadex G25 fine column (GE Healthcare) into 10 mM NH_4_HCO_3_, pH 7,9. For final endotoxin removal the protein solution was passed four times over a 1 mL Profos endotrap red column (Hyglos GmbH).

### Selection of human antibodies using phage display

ScFv were isolated in vitro from the human naïve phage libraries HAL7/8 by panning on decreasing amounts (10, 3 and 1 μg) of immobilized recombinant antigen 85 B according to [68].

### Production of soluble scFv antibodies for screening ELISA

The identification of monoclonal binders was performed as described in [69] with the following modifications: 96-well polypropylene (PP) micro titer plates (Greiner, Germany) containing 150 μL 2xYT-G/A (2xYT [67] supplemented with 100 mM glucose and 100 μg/mL ampicillin) were inoculated with colonies from the titration plate of the third panning round. Micro titer plates (MTP) were incubated overnight at 37°C and 850 rpm in a MTP shaker (PST-60HL-4, Lab4you, Austria). A volume of 180 μL 2xYT-G/A per well in PP-MTP was inoculated with 10 μL of the overnight culture and grown at 37°C and 850 rpm for two hours. Bacteria were harvested by centrifugation for 10 min at 3,220 × g and 180 μL supernatant were removed. The pellets were resuspended in 180 μL 2xYT supplemented with 50 mM sucrose and 100 μg/mL ampicillin + 50 μM IPTG and incubated at 30°C and 850 rpm overnight. Bacteria were pelleted by centrifugation for 15 min at 3,220 × g and 4°C. The scFv-containing supernatant was transferred to a new PP-MTP and stored at 4°C prior to analysis.

### Screening ELISA

96 wells of Microlon MTP (Greiner, Germany) were coated with 100 ng of recombinant 85 B in 100 μL PBS buffer pH 7.4 or BSA in 100 μL PBS buffer pH 7.4 (Carl Roth, Germany) as a negative control [67] overnight at 4°C. After coating, the wells were blocked with 300 μL PBST-B (PBS supplemented with 0.1% (w/v) Tween-20 and 1% (w/v) BSA) for 2 h at RT. This was followed by three washing steps with PBST0.05 (PBS supplemented with 0.05% (w/v) Tween-20). For identification of binders, supernatants containing monoclonal scFv were incubated in the antigen coated plates for 1.5 h at RT followed by three PBST washing cycles. Bound scFv were detected using mouse α-c-Myc-tag 9E10 (culture supernatant, 100 μL, 1:1,000 in PBST-B; 1.5 h at RT) followed by goat α-mouse IgG (Fc)-HRP (A0168, Sigma-Aldrich, Germany) (100 μL, 1:30,000 in PBST-B; 45 min at RT). After three washing steps with PBST0.05 the reactions were visualized with 100 μL 3,3’,5,5’-tetramethylbenzidine (TMB, Seramun, Germany) as a substrate. The staining reaction was terminated by addition of 100 μL 0.2 M H_2_SO_4_. Absorbance at 450 nm (620 nm reference) was measured using MRX ELISA reader (Dynatec, Germany).

### Antibody titration ELISA

For each antibody, 24 wells of Greiner Microlon MTP were coated with 100 ng antigen in PBS at 4°C overnight. BSA was coated as a negative control. After coating, the wells were blocked with PBST-B for 2 h at RT, followed by three washing steps with PBST0.05. Twelve dilutions of antibody (differing, depending on the antibody) in PBST-B were applied in duplicates on the antigen and BSA controls and incubated for 1.5 h at RT. Bound scFv-Fc were detected using goat α-human IgG (Fc)-HRP (A0170, Sigma-Aldrich, Germany) (100 μL, 1:130,000 in PBST-B; 45 min at RT). The assay was further processed as described above.

### Antigen titration ELISA

Antigen in PBS at twelve dilutions (1 μg/mL to 0.5 ng/mL) were coated in duplicates to wells of Greiner Microlon MTP at 4°C overnight. BSA coated wells were used as a negative control. After coating, the wells were blocked with PBST-B for 2 h at RT, followed by three washing steps with PBST. Antigen detection was carried out with a concentration of antibody at half maximal saturation (determined by antibody titration ELISA) in PBST-B for 1.5 h at RT followed by three PBST washing cycles. Bound scFv-Fc were detected using goat α-human IgG (Fc)-HRP (100 μL, 1:130,000 in PBST-B; 45 min at RT). The assay was further processed as described above.

### Direct ELISA

Analyte (antigen or antibody) was coated to the surface of Greiner Microlon 96 Well MTP at various concentrations in PBS buffer overnight at 4°C. Detection of antigen was carried out with an scFv-Fc antibody conjugated to HRP (by EZ-Link Plus Activated Peroxidase Kit, Pierce, Germany, according to the manufacturer’s instructions) diluted in PBST-B for 1 h at RT. Detection of antibody was carried out with Goat a-human IgG (Fc)-HRP diluted (100 μL, 1:130,000 in PBST-B) 45 min at RT. The assay was further processed as described above.

### Sandwich antigen titration

100 ng of capture antibodies were coated to the surface of 96 wells of Greiner Microlon MTP in PBS buffer overnight at 4°C. After coating, the wells were blocked with PBST-B for 2 h at RT, followed by three washing steps with PBST. Twelve dilutions of antigen in PBST-B were applied in duplicates onto the antibody coated wells and incubated for 1.5 h at RT, followed by three washing steps with PBST. Detection of bound antigen was performed with HRP conjugated scFv-Fc (exact dilutions described in results) in PBST-B for 1.5 h at RT, followed by three washing steps with PBST. The assay was further processed as described above.

### Epitope Mapping

The protein sequence of 85 B (Rv1886c, UniProt ID P0C5B9, sequence source: http://genolist.pasteur.fr/TubercuList/) was divided into overlapping peptide fragments, each consisting of 15 amino acids, with an offset of three amino acids. This array of peptides was synthesized by the SPOT technique [70,71] on an aminopegylated cellulose membrane (AIMS Scientific Products GmbH, Wolfenbüttel, Germany) as described previously [72]. Peptides are N-terminally acetylated and remain covalently attached to the membrane via their carboxy-terminus. The membrane bound peptide array was probed with the α-85 B antibodies for binding according to established procedures [30,72].

### Immunoblot

Proteins were separated on 12% SDS-PAGE [73,74] and semi-dry blotted onto polyvinylidene fluoride (PVDF) membranes (Carl Roth, Germany) according to the manufacturer’s instructions. Blocking was performed with MPBST (PBST0.1 supplemented with 2% (w/v) dry milk) for minimum 30 min at RT. Subsequent incubation with protein-specific antibody was carried out in MPBST for a minimum of 60 min at RT. Three washing steps with PBST0.1 for 5 min were performed. The PVDF membrane was incubated with a secondary antibody coupled to HRP in MPBST for at least 45 min at RT. The blot was washed another three times with PBST0.1 and then developed with TMB peroxidase membrane substrate (Seramun, Germany) until an adequate signal was obtained. Development was stopped by three short washing steps with deionized water.

### LFIA assembly + execution

5 – 40 μL cm^-1^ of colloidal gold-antibody conjugates were dispensed onto 8 mm glass fibre pads (Millipore, Germany) using the xyz-dispenser (Biodot, USA) and dried at 37°C. 1 μL cm^-1^ of different concentrations of capture antibody solution (sandwich assay) or antigen (direct assay) in PBS were dispensed in a line onto Unisart CN 95 nitrocellulose membranes (20 mm, Sartorius, Germany) that were already assembled onto 300 mm backing cards (Jieyi Biotechnology, China), and dried at 37°C. The glass fibre conjugate pads were laminated onto the backing cards, overlapping the nitrocellulose membrane at the connection point for ~ 0.5 – 1 mm. Then cellulose fibre pads (Millipore, Germany) were laminated onto the backing cards as sample and wicking pad, overlapping the nitrocellulose membrane at the connection point for ~ 0.5 – 1 mm. The assembled cards were cut into strips of 0.4 cm width with the CM4000 guillotine-cutter (Biodot) and the test strips were placed into Lateral Flow Strip Test (LFST) cassettes (Jieyi Biotechnology, China).

Several experiments were carried out to determine a procedure that would allow sensitive antigen detection and a low background combined with good feasibility (data not shown). The best option for a manageable LFIA was to add a diluent to the sample before applying it to the sample well. Therefore 1/3 sample volume (50 μL) of conjugation buffer (2 mM borax pH 8.8) enriched with 8% (w/v) BSA were mixed with 100 μL sample and applied to the LFIA. After 15 – 20 min results were optically evaluated.

### Yumab production + purification

All scFv isolated from antibody-phage display were subcloned into pCSE2.5-hIgG1-Fc-XP and produced as scFv-Fc (yumab) in HEK293-6E cells (National Research Council (NRC), Biotechnological Research Institute (BRI), Montreal, Canada). HEK293-6E cells were cultured in chemical defined F17 medium (Invitrogen, Life Technologies, Darmstadt, Germany) supplemented with 1 g/L pluronic F68 (Applichem, Darmstadt, Germany), 4 mM L-glutamine (PAA) and 25 mg/L G418 (PAA) as previously described [34]. DNA was transiently transfected into 25 mL HEK293-6E cells in 125 mL Erlenmeyer shake flasks. After 48 hours of cultivation at 110 rounds per minute (rpm) in a Minitron orbital shaker (Infors, Bottmingen, Switzerland) at 37°C and 5% CO_2_ atmosphere, one volume culture medium and a final concentration of 0.5% (w/v) of tryptone N1 (TN1, Organotechnie S.A.S., La Courneuve, France) were added. ScFv-Fc were purified via UNOsphere SUPrA column (Biorad, Munich, Germany) using a Profinia automate (Biorad, Hercules, California, USA), according to the manufacturer’s instructions.

### Analytical SEC

For analytical size exclusion chromatography (SEC) a Knauer PLATINblue HPLC Plus system (Knauer, Germany) with a Superdex 200 10/300 GL column (GE Healthcare, USA) was used according to the manufacturer’s instructions. The column was equilibrated with the Gel Filtration HMW/LMW Calibration Kits (GE Healthcare, USA) according to the manufacturer’s instructions.

### Tape Station

Protein solutions were analysed with the Screen Tape P200 Protein Standard Kit (Agilent, USA) under reducing conditions on a 2200 Tape Station system (Agilent, USA) according to the manufacturer’s instructions. The “P200 molecular weight standard” (Agilent) was used as a molecular marker.

## Competing interests

The authors have no competing financial or non-financial interests. This study was financially supported by the German Federal Ministry of Commerce, Grant No. KF 2584301. All aspects of cooperation and the generated results by the partner organisations have been mentioned in the cooperation agreement of the grant as required by the German Federal Ministry of Commerce. We did not receive any other financial or personal contribution by any other third party. Currently, there are no plans to file a patent. Following the rules of the German Federal Ministry of Commerce, LIONEX reserves its rights to develop any product based on the results or materials of this manuscript but agrees to provide the materials to research organisations only, exclusively for research use.

## Authors’ contributions

MF performed most of the experiments, participated in the design of the study, analysed data and drafted the manuscript. SK, SH, WP, RS, WO and RF performed some of the experiments, analysed data and provided material. SD participated in the design of the study and analysed data. MS and MH conceived the project and wrote the grant application, participated in the design of the study, analysed data and drafted the manuscript. All authors read and approved the final manuscript.

## Supplementary Material

Additional file 1: Figure S1 Screening ELISA for 85 B binding scFv in HAL7/8. Culture supernatants containing soluble scFv of 92 single clones (3. panning round) were screened for their ability to bind antigen (85 B) and BSA (negative control). On positions H9 and H12 an anti-lysozyme antibody was used on lysozyme as control for scFv production and ELISA detection system. Detection of bound scFv with mouse α-c-Myc-tag 9E10 IgG followed by goat α-mouse IgG(Fc)-HRP, development with TMB. Click here for file
