# Supplementary figures and images for: Novel human recombinant antibodies against Mycobacterium tuberculosis antigen 85B
Source: BMC Biotechnol. 2014 Jul 17;14:68. doi: 10.1186/1472-6750-14-68 (PMC4119940; doi:10.1186/1472-6750-14-68)

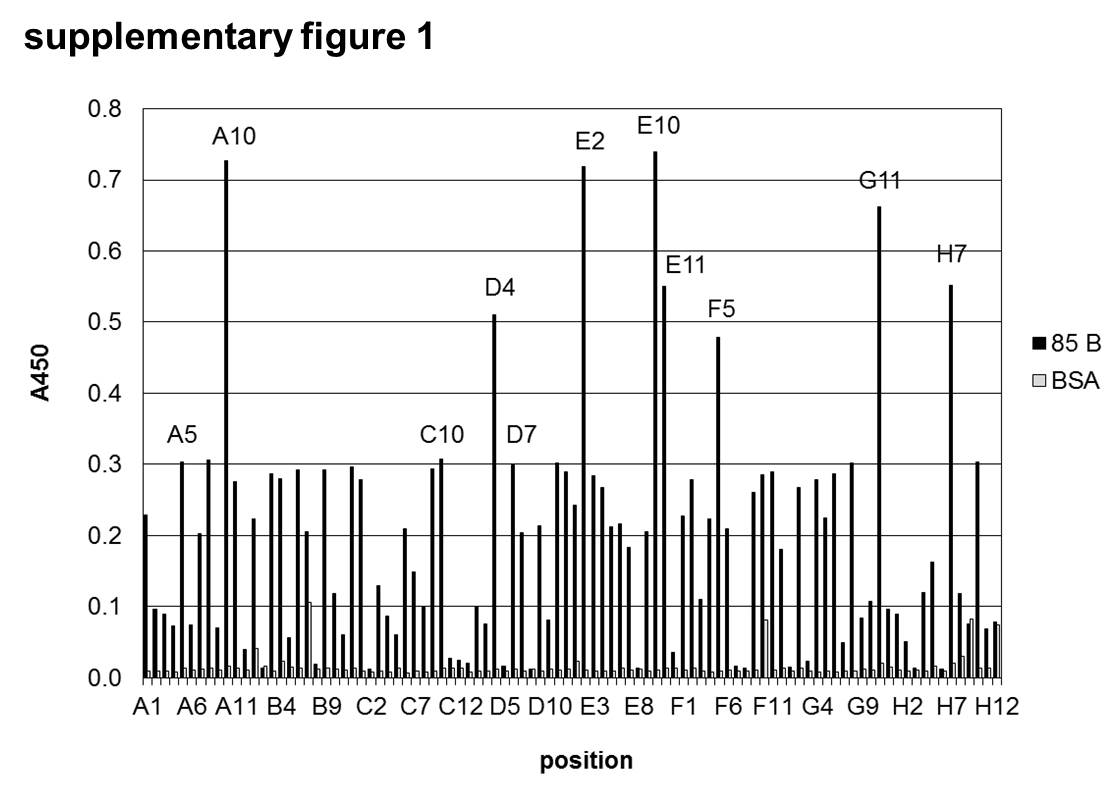

Supplement: Additional file 1: Figure S1 — Screening ELISA for 85 B binding scFv in HAL7/8. Culture supernatants containing soluble scFv of 92 single clones (3. panning round) were screened for their ability to bind antigen (85 B) and BSA (negative control). On positions H9 and H12 an anti-lysozyme antibody was used on lysozyme as control for scFv production and ELISA detection system. Detection of bound scFv with mouse α-c-Myc-tag 9E10 IgG followed by goat α-mouse IgG(Fc)-HRP, development with TMB. [file 1472-6750-14-68-S1.tiff]
